# RNA-Seq Reveals Spliceosome and Proteasome Genes as Most Consistent Transcripts in Human Cancer Cells

**DOI:** 10.1371/journal.pone.0072884

**Published:** 2013-09-17

**Authors:** Tara MacRae, Tobias Sargeant, Sébastien Lemieux, Josée Hébert, Éric Deneault, Guy Sauvageau

**Affiliations:** 1 Institute for Research in Immunology and Cancer (IRIC), Université de Montreal, Montreal, Quebec, Canada; 2 Molecular Medicine Division, The Walter and Eliza Hall Institute of Medical Research and Department of Medical Biology, The University of Melbourne, Parkville, Victoria, Australia; 3 Department of Computer Science and Operations Research, Université de Montreal, Montreal, Quebec, Canada; 4 Faculty of Medicine, Université de Montreal, Montreal, Quebec, Canada; 5 Division of Hematology, Maisonneuve-Rosemont Hospital, Montreal, Quebec, Canada; 6 Leukemia Cell Bank of Quebec, Maisonneuve-Rosemont Hospital, Montreal, Quebec, Canada; University of Pittsburgh, United States of America

## Abstract

Accurate quantification of gene expression by qRT-PCR relies on normalization against a consistently expressed control gene. However, control genes in common use often vary greatly between samples, especially in cancer. The advent of Next Generation Sequencing technology offers the possibility to better select control genes with the least cell to cell variability in steady state transcript levels. Here we analyze the transcriptomes of 55 leukemia samples to identify the most consistent genes. This list is enriched for components of the proteasome (ex. *PSMA1*) and spliceosome (ex. *SF3B2*), and also includes the translation initiation factor *EIF4H*, and many heterogeneous nuclear ribonucleoprotein genes (ex. *HNRNPL*). We have validated the consistency of our new control genes in 1933 cancer and normal tissues using publically available RNA-seq data, and their usefulness in qRT-PCR analysis is clearly demonstrated.

## Introduction

Normalization of measured levels of a gene of interest against a consistently expressed control gene is the most important action leading to accuracy in quantitative reverse-transcriptase PCR (qRT-PCR) experiments. However, while control gene levels can vary greatly depending on samples used, they are usually selected based solely on convention [1]–[6]. The advent of RNA-sequencing (RNA-seq) by Next Generation Sequencing (NGS) of thousands of transcriptomes of human samples offers new possibilities to identify and select control genes that show the lowest variation within the sample set for calculating relative gene expression using the ddCt method.

Leukemia and other cancer samples are prone to higher variability of gene expression compared to normal tissues due to clonal selection and genetic instability. Given the increased interest in expression profiling and identification of marker genes in cancer for personalized medicine, there is a clear need for optimal normalization of gene expression data by identifying control genes with the least possible variation.

Previous studies have been done in attempt to determine better endogenous control genes based on publically available microarray data [7], [8]. In such studies, microarray data from multiple tissues and conditions were analyzed in order to determine the genes whose expression varied the least, revealing mainly ribosomal protein coding genes. Next Generation Sequencing (NGS) technology has now replaced microarrays as the gold standard in global gene expression analysis. The analysis of gene expression by NGS has many advantages over microarrays, including a higher dynamic range and less susceptibility to technical variation [9]–[13]. Expression values typically used for RNA-seq are normalized for gene length and the total number of reads for each sample (Reads Per Kilobase of transcript per Million mapped reads: RPKM) [9], allowing for easy comparison between data sets. RNA-seq data mining therefore provides an ideal method to identify the most consistent genes for use as endogenous controls.

Here we exploit RNA-seq data from a panel of 55 Leukemia patient samples as well as 8 publically available RNA-seq data sets from The Cancer Genome Atlas (TCGA), (http://cancergenome.nih.gov/) to identify better endogenous control genes. We first demonstrate the variability of standard control genes as well as candidates suggested by microarray data analysis. We identify new control genes with lower variation across multiple cancer and normal tissue types, revealing primarily genes involved in RNA splicing and protein degradation processes. We then demonstrate the effectiveness of a selection of these genes in qRT-PCR. This new panel of highly consistent control genes will be of great use in future cancer research and disease monitoring.

## Materials and Methods

### Patient samples

Leukemia samples used in the Leucégène data set were collected by the Québec Leukemia Cell Bank with an informed written consent and approval of the project by the Research Ethics Board of the Maisonneuve-Rosemont Hospital and Université de Montréal as described [14]. Human cord blood samples were collected from healthy volunteers by Héma-Québec with an informed written consent and approval of the project by the Research Ethics Board of Ste. Justine Hospital and Université de Montréal.

### RNA-seq

RNA-seq was performed as described [14]. The data discussed in this publication have been deposited in NCBI's Gene Expression Omnibus [15] and are accessible through GEO Series accession number GSE48173 (http://www.ncbi.nlm.nih.gov/geo/query/acc.cgi?acc=GSE48173).

### qRT-PCR

Total RNA was isolated from leukemic and CD34+ cord blood cells using Trizol solution, according to the manufacturer's protocol (Invitrogen/Life Technologies, Burlington, ON, Canada). Human CD34+ cord blood cells were isolated from total cord blood using the RosetteSep Cord Blood CD34 Pre-enrichment kit, followed by the EasySep Human Cord Blood CD34+ Selection kit, according to manufacturer's guidelines (STEMCELL Technologies, Vancouver, BC, Canada), yielding 70–86% CD34+. CD34+ cord blood samples from five different individuals were immediately used for reverse transcription. Moreover, CD34+ cord blood samples from twelve additional individuals were sorted using FACS Aria cell sorter (Becton-Dickinson, San Jose, CA, USA) to keep only CD34_APC+/CD45RA_PE− cells (Antibodies: Becton-Dickinson, San Jose, CA, USA) before proceeding with reverse transcription. Reverse transcription of total RNA was performed using MMLV reverse transcriptase and random hexamers according to manufacturer's guidelines (Invitrogen/Life Technologies, Burlington, ON, Canada). Expression assays were performed to measure gene expression levels using 2× Fast Master Mix (Applied Biosystems/Life Technologies, Burlington, ON, Canada), standard primers (Invitrogen/Life Technologies, Burlington, ON, Canada) and a specific probe from the Universal Probe Library (Roche Diagnostics, Laval, QC, Canada). qRT-PCR reactions were done on the ABI 7900HT Fast Real-Time PCR System (Applied Biosystems/Life Technologies, Burlington, ON, Canada). For RQ (relative quantification) calculations, from a given test sample, the Ct (threshold cycle) values for each gene were normalized to the control gene (dCt = Ct Target – Ct Control) and compared to the mean dCT from the CD34+ cord blood sample (calibrator) using the ddCt method (ddCT = dCT Sample – dCt Calibrator; RQ = 2∧−ddCt). qRT-PCR cycling conditions were as follows: 2 minutes at 50°C and 10 minutes at 95°C, followed by 40 cycles of 15 seconds at 95°C and 1 minute at 59°C.

## Results

### Variability of commonly used control genes in RNA-seq data

For these studies, we made use of RNA-seq data obtained in our Leucégène project, which was acquired from a panel of 55 Leukemia patient samples (43 AML, 12 ALL) from The Québec Leukemia Cell Bank (BCLQ). We further analyzed RNA-seq data from various cancers and associated normal tissues, including AML, breast, lung, colon and kidney, all publically available from The Cancer Genome Atlas (TCGA). The combined TCGA data set represents data from a total of 1933 patients (207 normal tissue and 1726 cancer tissue samples) (**Table S1**).

To assess gene expression consistency, we examined the variability in RPKM values between different patient samples across a given RNA-seq data set. This was achieved by calculating the coefficient of variation (CV) and the maximum fold change (MFC) for each gene across multiple samples within each data set; where CV represents the standard deviation divided by the mean RPKM, and MFC represents the maximum RPKM divided by the minimum RPKM value.

We first analyzed the expression consistency of 19 commonly used control genes in the Leucégène and the combined TCGA data sets. Standard control genes were ranked from lowest to highest CV (**Table 1**). Using this approach, we found that the most consistent commonly used control gene, in both data sets, was TATA Binding Protein (*TBP*), yielding a CV equal to 22.8 or 44.9% and a MFC equal to 2.5 or 12.2, in Leucégène or combined TCGA data sets, respectively. Ableson (*ABL1*), a control gene commonly used for leukemia samples, yielded a slightly lower CV in the combined TCGA data set (39.8%), but had a high MFC (26.9). The majority of commonly used control genes exhibited variability, with CV values ranging from 27.2 to 69.1% in Leucégène (median CV = 42.6%), and 47.0 to 116.2% in the combined TCGA data (median CV = 61.4%). Not unexpectedly, we noted that the variability of the genes was higher in the combined TCGA data, which represents a more diverse collection of samples from five different cancer types and three different normal tissue types. This higher degree of variation in the combined TCGA data was more obvious in the MFC values, which are more greatly affected by extreme differences of expression in individual samples. MFC values ranged from 2.5 to 31.7 fold in Leucégène (median = 8.3), and 12.2 to 639.5 fold in the combined TCGA data (median = 84.0).

**Table 1:** Variability of most commonly used control genes in Leucégène and combined TCGA RNA-seq data sets.

		Leucégène			TCGA Combined		
rank	gene	mean	CV (%)	MFC	mean	CV (%)	MFC
1	TBP	8,1	22,8	2,5	6,7	44,9	12,2
2	YWHAZ	144,6	27,2	3,2	284,9	70,0	55,1
3	PGK1	189,9	28,4	3,4	212,6	62,0	31,0
4	LDHA	144,7	34,2	10,2	401,7	66,6	42,4
5	ALDOA	244,0	35,5	3,6	736,7	60,3	105,0
6	HPRT1	30,7	40,0	6,7	23,1	56,5	304,5
7	ABL1	17,0	40,1	5,7	13,9	39,8	26,9
8	SDHA	31,1	40,7	12,2	52,9	61,8	74,2
9	UBC	499,1	41,3	5,2	1260,8	47,0	102,0
10	GAPDH	2206,7	42,6	8,3	1954,8	70,7	60,7
11	ACTB	1617,9	48,7	5,4	2069,5	47,4	45,2
12	G6PD	43,5	52,6	6,9	23,9	106,7	639,5
13	VIM	1700,4	53,4	17,0	824,2	90,0	192,0
14	TUBA1A	251,0	53,8	8,4	148,7	55,6	64,6
15	PFKP	56,4	55,3	13,3	52,9	116,2	521,0
16	B2M	1798,6	55,5	13,9	2506,3	61,4	91,9
17	GUSB	45,6	55,9	10,6	44,7	61,2	84,0
18	PGAM1	12,9	65,5	14,4	125,4	60,1	95,4
19	HMBS	18,2	69,1	31,7	11,4	80,5	202,8

Mean relates to RPKM values within each data set. CV indicates the coefficient of variation and equals the standard deviation divided by the mean RPKM, expressed as a percentage. MFC, mean fold change, represents the maximum divided by minimum RPKM value of the data set. Rank is based on lowest to highest CV.

We further examined the expression consistency of 12 candidate control genes identified by de Jonge *et al.* [7] as being the most consistently expressed genes in a collection of microarray experiments. This gene list consists of 10 ribosomal protein coding genes, as well as *SRP14* and *OAZ1* (**Table 2**). Using the above approach, we found that the candidates identified from microarray data showed variability similar to those of the standard housekeeping genes, with a median CV equal to 48.5 or 51.6% and a median MFC equal to 8.3 or 44.5, in Leucégène or combined TCGA data sets, respectively. The most consistent gene from this list was Signal Recognition Particle 14 kDa (*SRP14*). Of note, while these genes presented similar variability in the Leucégène data set as compared to the commonly used control genes, they did prove to be slightly less variable in the combined TCGA data set. However, there was still significant variability within the TCGA data, which showed %CV values up to 82.0 for *RPS16*, and MFC values up to 1208.3 for *RPL9*.

**Table 2:** Variability of genes identified as stable in microarray experiments, in Leucégène and combined TCGA RNA-seq data sets.

		Leucégène			TCGA		
rank	gene	mean	CV (%)	MFC	mean	CV (%)	MFC
1	SRP14	132,2	24,9	3,2	145,6	31,8	10,9
2	RPL4	1276,1	40,4	5,6	734,0	44,3	51,0
3	RPL6	324,0	43,1	6,5	565,4	48,9	78,6
4	OAZ1	421,9	44,4	4,5	273,7	42,5	18,5
5	RPL22	156,6	45,4	9,0	192,4	39,2	25,8
6	RPL24	798,9	48,1	7,5	778,6	54,3	36,5
7	RPL27	1292,6	48,8	10,5	682,5	60,4	38,1
8	RPS13	935,8	55,0	8,8	662,8	47,7	29,3
9	RPS20	636,3	55,1	8,0	667,0	58,6	52,9
10	RPS29	559,0	56,2	8,7	490,9	65,8	100,3
11	RPS16	1104,4	61,6	9,2	794,2	82,0	192,1
12	RPL9	99,7	124,3	72,6	1007,0	66,3	1208,3

Genes identified by deJonge et al. [7] Mean relates to RPKM values within each data set. CV indicates the coefficient of variation and equals the standard deviation divided by the mean RPKM, expressed as a percentage. MFC (mean fold change) represents the maximum divided by minimum RPKM value of the data set. Rank is based on lowest to highest CV.

### Selection of improved control genes from Leucégène RNA-seq data

In order to identify improved control genes with the most consistent expression, we established cut-offs for %CV and MFC that were lower than the values obtained for the majority of commonly used control genes. Within the Leucégène data set, we analyzed the entire transcriptome of 21,892 genes and selected those which had a %CV less than 25 and a MFC less than 5, for two different ranges of expression: mean RPKM greater than or less than 100 (but greater than 25). These genes were then ranked from lowest to highest %CV (**Table 3**). Using these criteria, we identified 20 candidate control genes with mean RPKM levels greater than 100, and 99 candidate control genes with mean RPKM levels less than 100 (**Table 3** contains the best 20 genes; the full list is available in **Table S2**). The full list of 119 genes with their descriptions is available in **Table S4**. Of these, we selected 15 genes for validation based on their high ranking in the Leucégène data, as well as having relatively consistent expression in the various TCGA data sets (**Table S3**). The newly identified candidate control genes are: *HNRNPK, PCBP2, SLC25A3, GNB1, HNRNPL, SRP14* (RPKM>100); and *PSMD6, PSMA1, PSMF1, VPS4A, SF3B2, EIF4H, ZNF207, UBE2I* (RPKM<100). EIF4H had slightly higher expression in the various TCGA data sets, and was therefore included in the panel of genes with higher expression for subsequent analyses.

**Table 3:** Selection of candidate control genes based on Leucégène RNA-seq data.

Expression >100 RPKM					Expression <100 RPKM				
rank	gene	mean	CV (%)	MFC	rank	gene	mean	CV (%)	MFC
1	**HNRNPK**	220,4	16,5	2,0	1	MORF4L1	85,5	16,4	3,0
2	**PCBP2**	188,3	19,4	3,1	2	PSMD7	52,4	18,2	2,3
3	**SLC25A3**	149,1	19,8	2,5	3	**PSMD6**	45,8	18,4	2,2
4	**GNB1**	130,1	19,8	3,3	4	**PSMA1**	53,7	18,8	2,6
5	CCNI	176,2	20,9	2,9	5	SEC31A	36,0	18,8	2,5
6	HNRNPU	100,2	21,3	3,4	6	SRPR	50,7	18,9	2,6
7	**HNRNPL**	145,7	21,3	2,9	7	VCP	78,8	19,0	2,6
8	HNRNPD	124,4	22,4	3,1	8	**PSMF1**	27,1	19,2	3,4
9	CSDE1	137,1	22,5	3,6	9	MRFAP1	90,3	19,4	2,2
10	SRSF5	282,4	23,2	2,9	10	KHDRBS1	80,9	19,4	2,5
11	ATP5B	307,6	23,2	2,5	11	USP4	27,0	19,4	2,8
12	SSR2	136,7	23,7	3,4	12	DLST	34,9	19,6	2,6
13	MYL12B	137,9	23,8	3,5	13	**VPS4A**	35,1	19,8	2,7
14	HNRNPA2B1	238,2	23,9	3,6	14	SUPT6H	28,8	19,9	3,4
15	HNRNPC	125,2	24,3	3,3	15	**SF3B2**	82,9	19,9	2,3
16	ARF1	166,7	24,6	2,6	16	C1orf144	44,7	19,9	3,8
17	RHOA	307,0	24,6	2,9	17	NOL7	38,6	19,9	2,3
18	PSME1	147,7	24,7	3,7	18	**EIF4H**	95,8	20,0	3,3
19	DDX5	302,4	24,8	3,3	**43**	**ZNF207**	95,5	22,1	2,9
20	**SRP14**	132,2	24,9	3,2	**78**	**UBE2I**	56,1	24,2	4,1

Mean relates to RPKM values within each data set. CV indicates the coefficient of variation and equals the standard deviation divided by the mean RPKM, expressed as a percentage. MFC (mean fold change) represents the maximum divided by minimum RPKM value of the data set. Rank is based on lowest to highest CV. Criteria for gene selection were CV<25%, MFC<5 in Leucégène AML_ALL data. All genes fitting criteria for expression >100 RPKM shown; expression <100 RPKM table contains the 18 genes with the lowest CV in Leucégène AML_ALL data, as well two other selected candidates (full list of 99 genes available in Table S2). Genes listed in bold were selected for validation studies.

### Functional clustering of candidate control genes

We evaluated the functional classification of our entire list of 119 genes identified from the Leucégène data set using the DAVID algorithm [16], [17] (**Table S5**). Interestingly, a significant portion of these highly consistent genes fell into two main functional categories: RNA splicing/processing, with an enrichment score of 5.92 (ex. *SF3B2*); and proteasome/ubiquitin ligase activity, with an enrichment score of 5.76 (ex. *PSMA1*).

### Validation of new control genes in other RNA-seq cancer data sets

**Figure 1:**
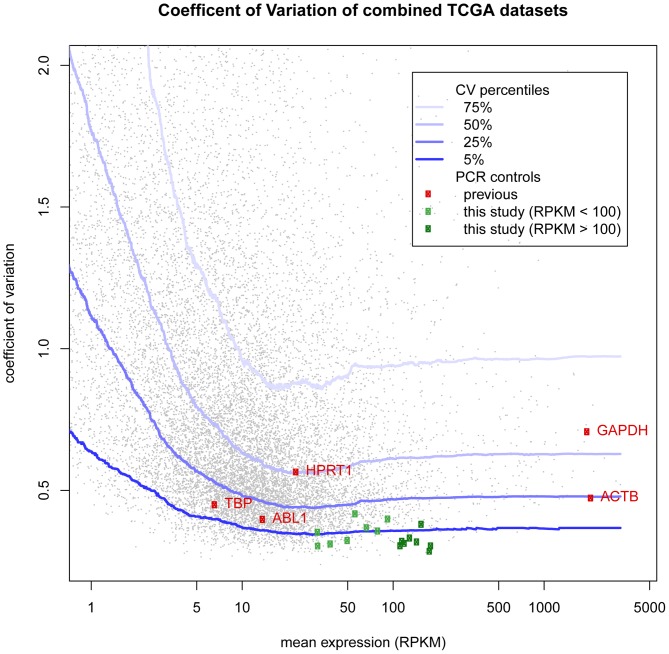
Distribution of coefficient of variation of control genes in relation to all genes in combined TCGA RNA-seq data. Mean expression represents the average of all RPKM values for a given gene across the combined TCGA data set (1933 samples). Coefficient of variation equals the standard deviation divided by the mean RPKM. Each dot represents a single gene: small grey dots represent entire transcriptome; dark and light green boxes represent new control genes with expression greater than or less than 100 RPKM, respectively; red boxes represent the indicated standard control genes. Curved blue lines represent the 5^th^, 25^th^, 50^th^ and 75^th^ quantiles of coefficient of variation for a given expression level (from darkest to lightest) computed over windows of 2000 ranked genes centered about a given mean RPKM value.

The expression consistency of the 15 candidate control genes was further examined in 8 different data sets from TCGA, representing 6 different cancer types and normal tissue samples, as well as in normal cord blood data obtained by Leucégène (**Table S1**). The 15 candidate control genes proved to be very consistently expressed in all 4 data sets of normal tissues, each yielding a CV less than or equal to 25%, and a MFC less than or equal to 10 (**Table S3**). Of note, the candidate genes showed highest consistency in the 17 CD34+ cord blood samples (enriched normal stem and progenitor cells), which each yielded CVs less than or equal to 15%, and MFCs less than 2. Within the tumor data sets, we observed more variability, with the highest CV being 42% for *SLC25A3* in kidney cancer, and the highest MFC being 24 for *SF3B2* in breast cancer. However, the majority of the candidate genes exhibited lower variability in all data sets as compared to the standard housekeeping genes. We determined a score for each candidate gene based on the number of data sets analyzed (10 total) in which the CV and MFC values complied with our initial selection criteria (CV<25%, MFC<5). The genes were then ranked according to this scoring system. We also calculated the expression variability of the candidate control genes using the combined TCGA data set (**Figure 1** **and** **Table 4**). As with the standard control genes, we did observe more variability compared to the individual data sets, reflecting the diversity of tissue types included. Nonetheless, all 15 of the candidate genes displayed consistency that was greater than the majority of the commonly used control genes. The CV values were all lower than that of *TBP*, however, *UBE2I* and *SF3B2* yielded CV values slightly higher than *ABL1*. Only *SF3B2* gave a MFC higher than that of *ABL1* (**Table 4**). The majority of the candidate genes had CV values in the lowest 5^th^ quantile and the remainder fell below the 25^th^ quantile, in contrast to the standard control genes, of which HPRT1 and GAPDH were actually more variable than half the genes present at similar expression levels (**Figure 1**).

**Table 4:** Variability of select candidate endogenous control genes in combined TCGA data sets.

Expression >100 RPKM							
rank	gene	mean	CV (%)	MFC	score	Chr	Gene Description
1	HNRNPL	116,3	32,0	8,8	10,0	19	heterogeneous nuclear ribonucleoprotein L
2	HNRNPK	177,0	28,4	8,8	7,5	9	heterogeneous nuclear ribonucleoprotein K
3	EIF4H	120,4	31,3	17,1	6,5	7	eukaryotic translation initiation factor 4H
4	PCBP2	180,3	30,4	9,8	6,0	12	poly(rC) binding protein 2
5	GNB1	113,2	30,4	16,9	6,0	1	guanine nucleotide binding protein beta
6	SRP14	145,6	31,8	10,9	6,0	15	signal recognition particle 14 kDa
7	SLC25A3	156,1	38,0	16,0	6,0	12	solute carrier family 25 member 3

Expression <100 RPKM							
rank	gene	mean	CV (%)	MFC	score	Chr	Gene Description
1	ZNF207	50,6	32,3	10,7	9,0	17	zinc finger protein 207
2	UBE2I	57,0	41,7	12,6	7,5	16	ubiquitin-conjugating enzyme E2L
3	VPS4A	32,3	30,3	17,4	7,0	16	vacuolar protein sorting 4 homolog A
4	PSMF1	39,0	31,1	19,1	6,5	20	proteasome inhibitor subunit 1 (PI31)
5	PSMA1	67,8	36,8	18,0	6,5	11	proteasome subunit, alpha type, 1
6	SF3B2	93,7	39,9	34,3	6,5	11	splicing factor 3b, subunit 2
7	SRSF9	80,3	35,6	23,8	6,0	12	serine/arginine-rich splicing factor 9
8	PSMD6	32,1	35,2	16,6	5,5	3	proteasome 26S subunit, non-ATPase, 6

Mean relates to RPKM values within each data set. CV indicates the coefficient of variation and equals the standard deviation divided by the mean RPKM, expressed as a percentage. MFC, mean fold change, represents the maximum divided by minimum RPKM value of the data set. Score represents the sum of the number of datasets (out of 10 total) which have a CV<25% and MFC<5 for each gene, divided by 2. Chr indicates the chromosome number. Rank is based on highest to lowest score.

Overall, the 15 newly selected control genes display a greater degree of consistency in gene expression compared to the commonly used control genes, as determined by RNA-seq. The highest ranking genes, as determined by having low coefficient of variation (CV) and maximum fold change (MFC) values in the most data sets analyzed are: HNRNPL and ZNF207, with high and medium expression ranges, respectively.

### QPCR validation of new control genes

**Figure 2:**
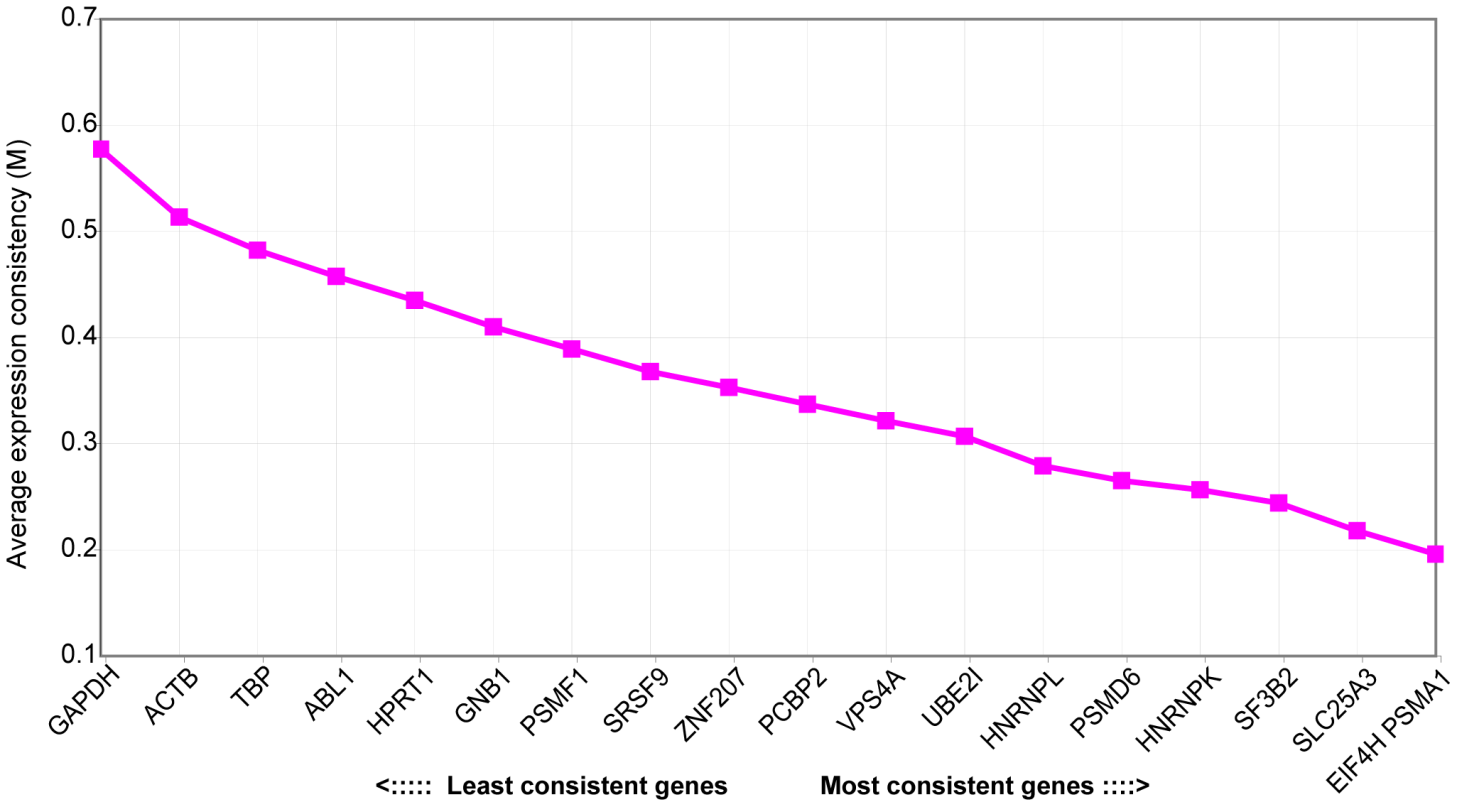
Average expression consistency of control genes in qRT-PCR. Average expression consistency (M) was calculated with the GeNorm algorithm [18] based on qRT-PCR for the indicated control gene on a panel of 14 leukemia samples and one cord blood sample. Lower M values relate to genes which proved to have more consistent expression levels across the samples used.

In order to assess the effectiveness of the newly identified control genes for quantitative RT-PCR (qRT-PCR) analysis, we developed assays for the candidates using the Universal Probe Library (Roche) (**Table S6**). New assays were designed to span intron boundaries, and tested for optimal efficiency by standard curve analysis. *SRP14* was excluded due to the inability to design an intron spanning assay. qRT-PCR was performed for each of the 14 new genes, as well as for 5 standard control genes (*GAPDH, ACTB, TBP, HPRT1, ABL1*), on cDNA from a panel of 14 leukemia samples (10 AML, 4 ALL) plus one CD34+ cord blood sample (using equal amounts of RNA). The average expression consistency (M) of each gene was calculated using the GeNorm algorithm [18] (**Figure 2**). By qRT-PCR, all 14 of the newly identified control genes had lower M values than the standard control genes, confirming that they were more consistently expressed in the leukemia samples, in agreement with the RNA-seq data, with *EIF4H* and *PSMA1* being the most consistent in this experimental condition.

**Figure 3:**
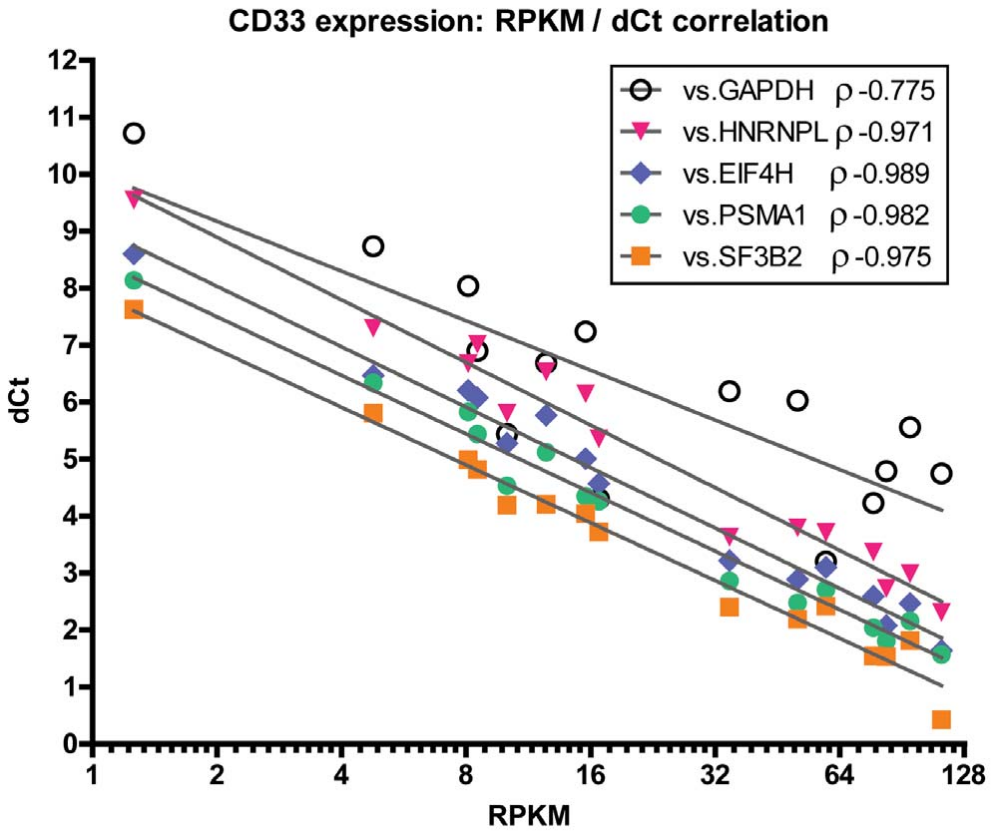
Correlation between RPKM and delta Ct of *CD33* calculated with different control genes. dCt represents the difference between the Ct value of *CD33* and that of the indicated control gene, for a given leukemic sample, measured by qRT-PCR. RPKM is plotted on a log-2 scale and represents the Reads Per Kilobase of transcript per Million mapped reads obtained for each leukemic sample by RNA-seq. ρ represents the Spearman correlation coefficient between the RPKM and the dCt obtained with the indicated control gene.

Although it is widely presumed that RNA-seq data correlates well with qRT-PCR data, there is little evidence available to address this topic. We therefore assessed the expression of *CD33* and *FLT3* (data not shown) in the same 15 leukemia and cord blood samples in order to demonstrate correlation between the RPKM and delta Ct (dCt) values for this gene. These two genes were selected due to their known variability of expression in leukemia. The delta Ct values for each sample were calculated using either a standard control gene (*GAPDH*), or a newly identified control gene (*HNRNPL, EIF4H, PSMA1, or SF3B2*). Spearman correlation analysis of *CD33* expression data demonstrated high correlation between RPKM and dCt (ρ = −0.9714 to −0.9893 for *EIF4H*), except when *GAPDH* was used as the control gene (ρ = −0.775) (**Figure 3**). Analysis with *FLT3* showed similar correlation. The lower degree of correlation between RPKM and dCt when using *GAPDH* as a control gene demonstrates the importance of proper control gene selection in qRT-PCR experiments.

**Figure 4:**
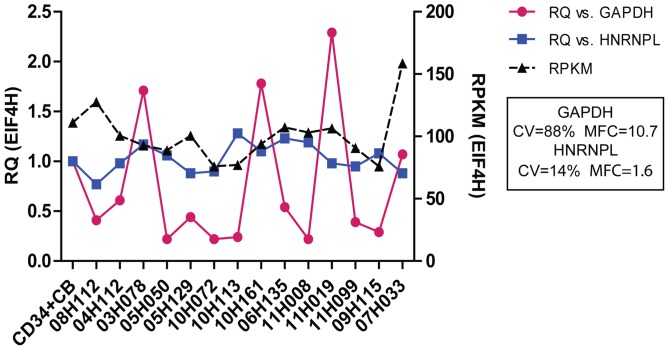
Comparison of *EIF4H* gene expression values calculated with *GAPDH* or *HNRNPL*. RQ represents relative quantification of *EIF4H* determined by qRT-PCR, calculated using the ddCt method with either *GAPDH* or *HNRNPL* as the control gene, relative to the CD34+ cord blood (CB) sample. The X axis indicates the leukemic sample ID. CV (expressed as a percentage) indicates the coefficient of variation and equals the standard deviation divided by the mean RQ of CD33 calculated using the indicated control gene. MFC (mean fold change) represents the maximum divided by minimum RQ value.

To further address the importance of proper control gene selection in qRT-PCR analysis, we calculated the relative quantification (RQ) values for a consistently expressed gene (*EIF4H*), using either *GAPDH* or *HNRNPL* for normalization (**Figure 4**). As expected, the RQ of *EIF4H* varied very little between leukemia samples when *HNRNPL* was used as the control gene (CV = 14%; MFC = 1.6). However, RQ values of the same samples calculated using *GAPDH* varied as much as 10.7 fold, with RQ values ranging from 0.22 to 2.29 (CV = 88%). Normalization with *GAPDH* resulted in up to a 5.3 fold difference in *EIF4H* expression within individual samples, as compared to *HNRNPL* normalization. These findings highlight the importance of using more consistent control genes as identified in this study in qRT-PCR analysis, and further validate our newly identified control genes.

## Discussion

Evaluation of gene expression by quantitative RT-PCR (qRT-PCR) relies on normalization with an endogenous control gene, resulting in relative quantification of the gene of interest. Most researchers use only a single control gene, the selection of which is often based solely on convention [3], [6]. The control genes most commonly used were originally selected due to their high expression levels in all tissues rather than their low variability among tissues [6]. However, numerous studies have shown that these genes can vary considerably [1]–[5], thus casting doubt on the accuracy of relative quantification values.

While many studies have been done in attempts to determine better methods for normalization of gene expression [6], [18]–[20], most researchers still choose to use the ddCt method with one or two control genes, without proper validation of those controls. There have been relatively few studies that aimed to identify new control genes whose expression levels are more consistent than those in common use, such as is presented here. A couple of studies which have been done with this shared goal relied on microarray data meta-analysis [7], [8], while our study uses next generation sequencing data. Both of these studies identified mainly ribosomal protein (*RP*) coding genes, whereas our analysis did not reveal any genes from this family. In fact, we show here that the specific RP genes outlined by de Jonge *et al.* [7] are similar to that of the standard control genes with respect to their variability in gene expression, as determined by RNA-seq. *RP* genes represent the most highly expressed group of genes (approximately 50% of the top 100 most highly expressed genes in RNA-seq data analyzed, data not shown). Therefore, one possible explanation for the discrepancy between analyses performed on microarray vs. RNA-seq data could be that saturation of the fluorescence signal in microarrays has lead to a false impression of consistency. While the RPKM calculation of short genes (such as *RP* genes) may be prone to higher technical variability than long genes, at high expression levels this effect is small, and the CV is dominated by biological variation. In fact, CV values for *RP* genes in the combined TCGA dataset showed a fair spread at all expression levels (data not shown), implying that there is no bias for RP genes in the RNA-seq data.

RNA-seq analysis has many advantages over microarrays for the analysis of global gene expression. Most notably, because RNA-seq reads are digital rather than analog, there is very low background signal, and virtually no upper limit for detection, resulting in a much larger dynamic range [9]–[13], [21]. Studies have revealed a higher degree of technical reproducibility with RNA-seq over microarrays [9], [10], and that RNA-seq expression levels correlate better with qRT-PCR data, regardless of the sequencing platform used [21]. Microarray data is susceptible to errors resulting from hybridization artifacts, saturation of fluorescent signal, and requires complicated normalization [10]–[12]. RNA-seq circumvents these issues; however, other potential sources for errors exist, such as gene length bias, bias in sequencing of GC rich regions, technical issues in library preparation, or errors in read mapping [10], [12]. RNA-seq is also not limited by prior knowledge of the transcriptome being studied, allowing for the identification of novel transcripts and SNPs.

Here we identify a total of 119 genes whose expression is more consistent than the commonly used control genes across a panel of 55 leukemia samples, as determined by RNA-seq. Functional classification of these by DAVID revealed two main enrichment clusters: genes involved in the proteasome/ubiquitin degradation pathways (ex. *PSMA1, PSMF1, UBE2I*), and genes involved in RNA splicing and processing (ex. *SF3B2*, *SRSF9*). In addition to these functional clusters, we found 12 genes involved in transcription and 7 involved in translation (ex. *EIF4H*). A prominent group of genes identified (n = 8) are the heterogeneous nuclear ribonucleoproteins (ex. *HNRNPL, HNRNPK*), some of which are also involved in the above cellular processes. Of note, the study by Popovici *et al.* [8] also identified two *HNRNP* genes, one proteasome subunit gene, *Ubiquitin B* and *C*, and *EIF4H* as having highly consistent expression across ten breast cancer microarray data sets. In concordance with the studies from de Jonge and Popovici, we also identified *SRP14* as a good control gene. Although *SRP14* was a strong candidate, we were unable to design an intron-spanning qRT-PCR assay for it, and it was therefore not included in our validation experiments.

Of the 119 genes selected from the leukemia RNA-seq data, 14 were selected based on their consistency in other RNA-seq data sets (TCGA) for validation by qRT-PCR. This was essential to account for potential biases inherent to the RNA-seq procedure, such as the selection of poly-A+ RNA, cDNA fragmentation and library preparation, as well as potential biases introduced bioinformatically [12]. Nonetheless, we confirmed that all 14 genes tested proved to be more consistent by qRT-PCR in a selection of 14 leukemia samples than the standard control genes. Furthermore, we have shown that RPKM values obtained by RNA-seq correlate well with dCt values obtained by qRT-PCR, and that this correlation is dependent on the control gene used for dCt calculation. We also clearly demonstrate the impact of proper control gene selection in qRT-PCR experiments, since the calculation of relative quantification values (RQ) of *EIF4H* (a highly consistent gene by RNA-seq) varied significantly when *GAPDH* was used as opposed to our new control, *HNRNPL*.

Quantitative RT-PCR is increasingly used for diagnostic and disease monitoring purposes, such as the evaluation of minimal residual disease (MRD) in leukemia. Given the highly sensitive nature of these assays, it is of utmost importance to use the best possible control gene for normalization. Ableson (*ABL1*) has previously been shown to be the most consistent control gene tested for MRD detection [22]. However, the control genes identified here all proved to be more consistent than *ABL1* both by RNA-seq and qRT-PCR of leukemia samples, making them ideal candidates for use in MRD.

Although the control genes presented here were initially selected due to their consistency in leukemia samples, we have selected those which were also relatively consistent in other cancer types as well as associated normal samples, thus potentially extending their utility as general control genes for most human tissues. Based on our validation studies, we expect that our new controls will outperform the standard control genes in a wide variety of sample types. However, for other cancer types, better control genes may exist, which could be determined using the same approach used here. It will be important for researchers to validate these new controls before their use with more diverse tissue types.

It would be interesting to further assess the consistency of our new control genes in mouse or other model organisms. To date, there is less publically available RNA-seq data available for non-human cell types. Although groups such as The Encyclopedia of DNA Elements (ENCODE) Consortium provide easy access to a wealth of NGS data with many mouse cell types represented [23], most RNA-seq experiments have only 2–3 replicates, in contrast to the large number of human samples used in The Cancer Genome Atlas (TCGA) data sets. As NGS technology becomes more widely available, it may soon be feasible to assess the consistency of these control genes in other organisms.

In conclusion, we have made use of RNA-seq data to identify 14 new control genes with consistent expression in various cancer types. These genes, including *HNRNPL*, *EIF4H* and *PSMA1*, were validated by qRT-PCR for use as control genes in leukemia.

## Supporting Information

Table S1 The RNA-seq data sets analyzed in this study. Leucégène, RNA-seq data generated in collaboration between the Leukemia Cell Bank of Quebec and The Genomic Core Facility at Institute for Research in Immunology and Cancer (IRIC); TCGA, The Cancer Genome Atlas Data Portal (http://cancergenome.nih.gov/).
(XLSX)

Table S2 Candidate control genes identified in the Leucégène data sets. Only genes for which the abundance of transcript levels exceeded 100 RPKM (Reads Per Kilobase of transcript per Million mapped reads) were included.
(XLSX)

Table S3 Variability of the selected candidate endogenous control genes in normal hematopoietic cells and in TCGA data sets. CV, coefficient of variation; MFC, maximum –fold change.
(XLSX)

Table S4 Description of candidate endogenous control gene function. Known function(s) of genes were retrieved from www.uniprot.org.
(XLSX)

Table S5 Functional classification of candidate genes. Enrichments in various annotation clusters in the Leucégène data set were determined using the DAVID functional annotation tool (http://david.abcc.ncifcrf.gov).
(XLSX)

Table S6 Primers and probes used for Q-PCR assays.
(XLSX)
